# Efficacy of acupuncture on pelvic pain in patients with endometriosis: study protocol for a randomized, single-blind, multi-center, placebo-controlled trial

**DOI:** 10.1186/s13063-018-2684-6

**Published:** 2018-06-07

**Authors:** Ruining Liang, Peishuang Li, Xuemei Peng, Ling Xu, Pei Fan, Jiahua Peng, Xu Zhou, Chunlin Xiao, Miao Jiang

**Affiliations:** 1 Institute of Obstetrics and Gynecology, Jiangxi University of Traditional Chinese Medicine, Nanchang, China; 2 Department of Obstetrics and Gynecology, the Second Affiliated Hospital, Jiangxi University of Traditional Chinese Medicine, Nanchang, China; 3 Department of Traditional Chinese Medicine, Jiangxi University of Traditional Chinese Medicine, Nanchang, China; 4 Department of Gynecology, the Affiliated Hospital, Jiangxi University of Traditional Chinese Medicine, Nanchang, China; 5 Evidence-based Medical Center, School of Basic Medical Sciences, Jiangxi University of Traditional Chinese Medicine, Nanchang, China; 6 Department of Characteristic Therapy with Traditional Chinese Medicine, the Affiliated Hospital, Jiangxi University of Traditional Chinese Medicine, Nanchang, China

**Keywords:** Acupuncture, Endometriosis, Pelvic pain, Study protocol

## Abstract

**Background:**

Endometriosis is a chronic gynecological disease that is characterized by the presence of endometrial tissue outside the uterine cavity. The main symptoms include dysmenorrhea, dyspareunia, chronic pelvic pain, and infertility. These symptoms impair the lives of most of the women suffering from the disease. Surgical resection of endometriotic lesions is an effective means of treating dysmenorrhea, but the risk of recurrence is high. Western medicine has limited use for treating it due to side effects and ineffectiveness. The purpose of this study is to verify the effectiveness and safety of acupuncture.

**Methods/design:**

This trial will be carried out in four parts. A total of 106 eligible patients with pelvic pain related to endometriosis will be randomly assigned into two groups, in a 1:1 ratio, as the treatment group or the control group. The participants assigned to the treatment group will be treated with acupuncture treatment at *Guanyuan* (CV4), *Sanyinjiao* (SP6), *Taichong* (LR3), *Zhaohai* (KI6) and *Qichong* (ST30) while the control group will receive acupuncture at non-acupoints. The trial will include three menstrual cycles of treatment and three menstrual cycles of follow-up. The primary outcome is pelvic pain that will be assessed by means of a 10-cm visual analog scale (VAS). At each stage, we will evaluate the safety of the acupuncture treatment.

**Discussion:**

The study will compare the effectiveness and safety of acupuncture with comfort needles on pelvic pain related to endometriosis in the hope of providing significant evidence for using acupuncture on pelvic pain related to endometriosis.

**Trial registration:**

ClinicalTrials.gov, ID: NCT03125304. Registered on 30 April 2017.

**Electronic supplementary material:**

The online version of this article (10.1186/s13063-018-2684-6) contains supplementary material, which is available to authorized users.

## Background

Endometriosis is a chronic gynecological disease characterized by endometrial tissue being present outside the uterine cavity. It occurs in approximately 10% of adolescent girls and women of reproductive age [1]. The main symptom is pelvic pain which includes dysmenorrhea, intermenstrual pain and dyspareunia, which seriously impairs quality of life [2].

The pathogenesis of pain associated with endometriosis is not clear but may be related to inflammation from endometrial lesions, peripheral and central sensitization [3]. Surgical interventions can greatly alleviate pain, but the risk of recurrence is high. The postoperative recurrence rate of endometriosis is 40–50% in 5 years [4]. Drug treatments include oral contraceptives, progesterone, androgen agents and gonadotropin-releasing hormone agonists, but their use is limited because of unacceptable side effects. In a survey involving 1160 women with endometriosis, drug treatment cessation was mostly related to side effects and drug ineffectiveness [5].

Acupuncture, an important component of traditional Chinese medicine, plays a crucial role in disease treatment. Acupuncture is also a popular means of analgesia in traditional Chinese medicine. The analgesic principle of acupuncture is based on the holistic theory of *zang-fu* organs and meridians [6]. Traditional Chinese medicine holds that pain is caused by the stagnation of blood or *Qi* in the uterus. Acupuncture can improve blood and *Qi* circulation, depending on the conduction of meridian acupoints and the function of the central and peripheral nervous system. When the flow of *Qi* and blood is unobstructed, pain can lessen [7]. Some systematic reviews show clinical value in acupuncture for pain [8–10].

One systematic review supports acupuncture for pain relief in endometriosis. However, the evidence for the effectiveness of acupuncture is limited because only a single study was included in this review [11]. Fourteen systematic reviews were included in a Cochrane review, assessing pain relief following intervention with gonadotropin-releasing hormone (GnRH) analogs, ovulation suppression, non-steroidal anti-inflammatory drugs (NSAIDS), surgical interventions, postsurgical medical interventions, alternative medicine (auricular acupuncture compared to Chinese herbal medicine) and anti-TNF-α drugs. The quality of evidence for specific comparisons ranged from very low to moderate. There were limitations in the evidence including a risk of bias in the primary studies, inconsistency between the studies and imprecision in effect estimates [12]. Although acupuncture for pelvic pain in endometriosis has its clinical value, the supporting evidence is limited. Therefore, a randomized, multi-center controlled trial is necessary to provide quality evidence that can guide clinical practice.

### Objective

The purpose of the present study is to demonstrate the efficacy in pain relief and safety of acupuncture in patients with pelvic pain related to endometriosis.

## Methods/design

### Study design

This randomized trial will be conducted in four centers: the Second Affiliated Hospital of Jiangxi University of Traditional Chinese Medicine, the Affiliated Hospital of Jiangxi University of Traditional Chinese Medicine, the Qionghai Hospital of Traditional Chinese Medicine and the Ganzhou Maternal and Child Health Care. A total of 106 patients diagnosed with pelvic pain related to endometriosis will be enrolled in this trial (Additional file 1).

### Participants

**Fig. 1:**
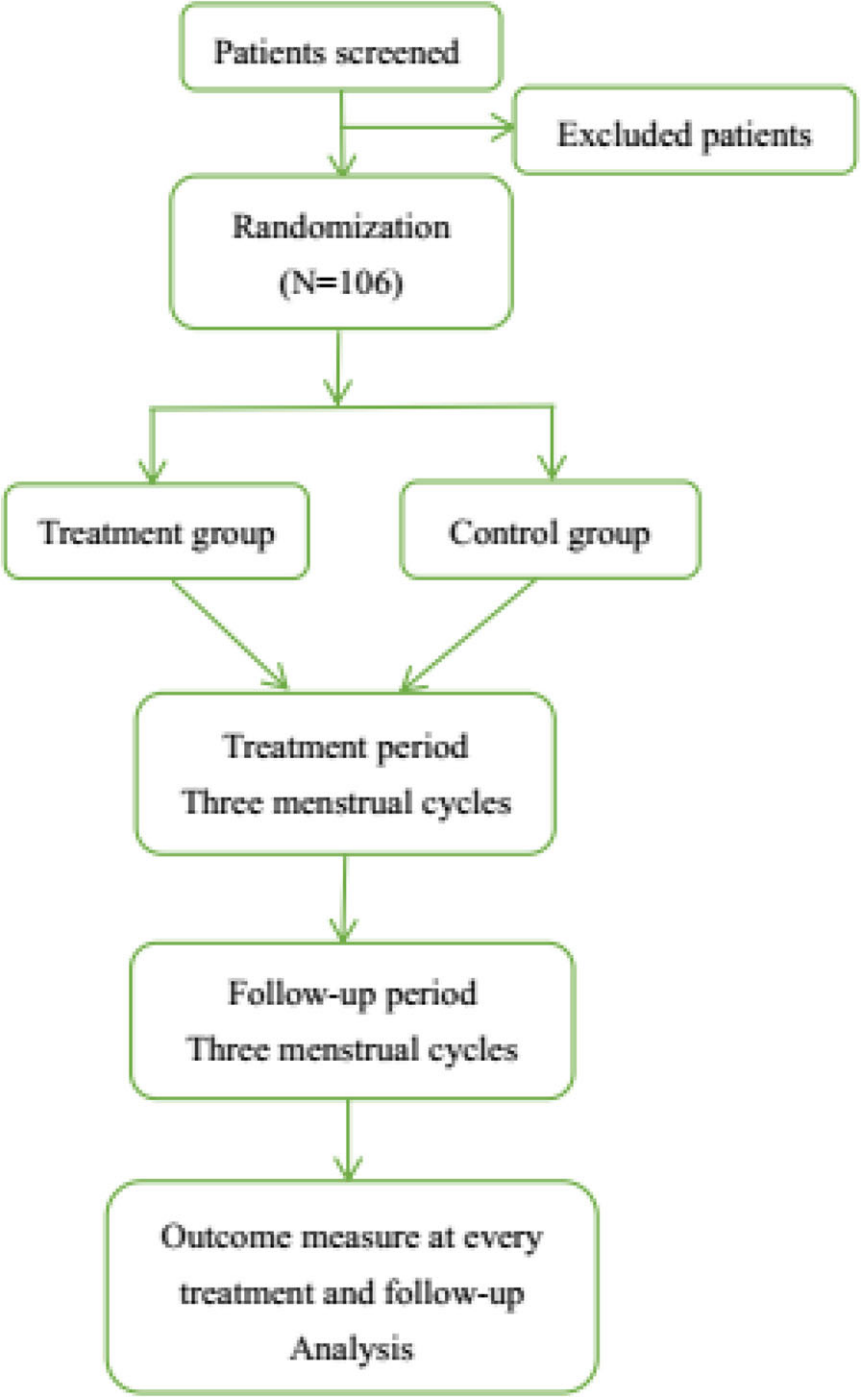
Participant flow diagram

Participants will be recruited only if they satisfy all of the inclusion criteria and do not meet any of the exclusion criteria. See Fig. 1 for the participant flow diagram.

#### Inclusion criteria

1. Women aged between 20 and 40 years
2. Endometriosis diagnosed according to the Endometriosis Consensus Guidelines (Chinese Medical Association, 2015): (1) symptomatic endometriosis detected by laparoscopy or laparotomy or (2) ovarian endometrioma detected by ultrasound or magnetic resonance imaging with pelvic pain
3. Patients willing to receive acupuncture for pain relief
4. Pelvic pain score equal to or above 4 cm on the Visual Analog Scale (VAS)
5. Regular menstrual cycle
6. Signed informed consent

#### Exclusion criteria

1. Pelvic ultrasound demonstrates that the endometrial cyst measures more than 5 cm in diameter and surgery is indicated
2. Imaging examination suspects pelvic genital malignancy
3. Cancer antigen-125 level > 200 IU/L
4. Pelvic infectious disease
5. Other severe disorders in the heart, lung, liver, kidney, or mental disorders causing inability to cooperate in the study
6. Patients who have received any other treatment for endometriosis that may affect the observation of the curative effects of acupuncture over 3 months
7. Pregnancy or breastfeeding
8. Refusal to be randomized

### Interventions

This study has two phases. The first phase will include 3 months of acupuncture treatment, and the second phase will include 3 months of follow-up without treatment. Patients will be randomly assigned to one of two groups in a 1:1 ratio, either the treatment group or the control group. Then, acupuncture treatment will start and last for 3 months following by 3 months of follow-up.

The whole acupuncture treatment will be carried out by two experienced acupuncturists who have received a license from the Ministry of Health of China and have practiced clinically for more than 5 years. Every acupuncture session will be once a day, three times a week and last for 30 min and only start from 7 days before the expected menstrual onset. During the menstrual period, they will receive acupuncture every day when they have endometriosis-associated pelvic pain.

#### Treatment group

The design of the treatment group is guided by the theory of Traditional Chinese Medicine. Disposable, sterile, stainless-steel needles, 0.30 × 40 mm, will be inserted into the acupoints on the abdomen and legs for 15 to 35 mm. The participants assigned to the treatment group will receive acupuncture at bilateral *Sanyinjiao* (SP6), *Zhaohai* (KI6), *Taichong* (LR3), *Qichong* (ST30) and at *Guanyuan* (CV4) for a total of nine needles see (Table 1) [13]. After insertion, all acupuncture needles will be stimulated manually by rotating the needle quickly with the thumb and forefinger to evoke the needle sensation (*De Qi*) once every 10 min.

**Table 1:** Acupuncture points

Point	Localization	Muscle	Muscle innervation
*Guanyuan* (CV4)	3 cun caudal to umbilicus	Fibrous tissue, linea alba	Th12
*Sanyinjiao* (SP6)	3 cun proximal to the medial malleolus	Musculus flexor digitorum longus, tibialis posterior	L4–5,S1–2
*Taichong* (LR3)	Depression between the first and second metatarsals	Musculi interossei dorsales	S2–3
*Zhaohai* (KI6)	Depression below the medial malleolus	Abductor of toes	L4–5, S1–2
*Qichong* (ST30)	5 cun caudal to umbilicus, 2 cun proximal to the anterior median line	Obliquus internus abdominis	Th12, L1

#### Control group

The control group will receive acupuncture at non-acupoints. Disposable, sterile, stainless-steel needles will be inserted superficially to a depth of < 5 mm on each shoulder and upper arm, without any stimulation.

### Primary outcome

#### Pelvic pain

The primary outcome is pelvic pain that include dysmenorrhea, intermenstrual pain and dyspareunia. They will be assessed on a VAS scale [14]. On the VAS scale, a 100-mm horizontal line from no pain on the far left to the worst possible pain on the far right is scored by determining the distance from the far left to the edge of the mark made by the patient. The distance represents pain intensity. The VAS will be assessed at baseline, the first, second and third menstrual cycles and the follow-up period. See Table 2 for the study design schedule.

**Table 2:** Study design schedule

Period	Inclusion	Treatment			Follow-up		
Visit	1	2	3	4	5	6	7
Menstrual cycle	0 month	1	2	3	4	5	6
Patients	√						
Inclusion and exclusion criteria	√						
Informed consent	√						
Physical examination	√			√			
Medical history	√						
Comorbidities	√	√	√	√	√	√	√
VAS	√	√	√	√	√	√	√
EHP-30	√			√			√
MPI	√			√			√
BDI and POMS	√			√			√
PGIC	√			√			√
Pain diary	√	√	√	√	√	√	√
Patient compliance				√			√
Reasons for dropout or withdrawal				√			√
Adverse events				√			
Safety evaluation							√

*BDI* Beck Depression Inventory, *EHP* Endometriosis-specific quality of life, *MPI* Multidimensional Pain Inventory, *PGIC* Patient Global Impression of Change, *POMS* Profile of Mood States, *VAS* Visual Analog Scale

### Secondary outcomes

#### Endometriosis-specific quality of life

The Endometriosis Health Profile − 30 (EHP-30) [15] will be used to evaluate the effects of endometriosis on patients’ lives. This questionnaire includes two parts. The first part is the core questionnaire applicable to all patients with endometriosis, which includes five dimensions and 30 items. The second part of the modular questionnaire includes 23 items and six dimensions; each dimension is specific to the endometriosis patient: employment, relationship with children, sex, feelings about the medical profession, treatment and infertility. Each item is rated at five grades from 0 as “never” to 4 as “always.” The EHP-30 will be assessed at the baseline period, the third menstrual cycle during treatment and the third menstrual cycle in the follow-up period.

#### Physical functioning

Physical functioning will be assessed by the Multidimensional Pain Inventory (MPI) [16]. This questionnaire includes three parts. The first part is the influence of pain on patients’ daily lives, the second part is the response of patients’ significant other to patients regarding pain, and the third part is the ability of patients to participate in daily activities. Each item is scored at seven grades from 0 to 6. The MPI will be assessed at the baseline period, the third menstrual cycle during treatment and the third menstrual cycle in the follow-up period.

#### Emotional functioning

Emotional functioning will be evaluated by the Beck Depression Inventory (BDI) [17] and Profile of Mood States (POMS) [18]. The BDI consists of 13 items of emotional, behavioral and somatic symptoms. Each item is scored from 0 to 3 and measure sadness, pessimism, sense of failure, lack of satisfaction, guilty feelings, sense of punishment, self accusations, social withdrawal, indecisiveness, body image, work inhibition, fatigability and loss of appetite. The POMS is a psychological rating scale used to assess distinct mood states and measure six different dimensions of mood swings. These include: tension, depression, anger, vigor, fatigue and confusion. A 5-point scale ranging from “not at all” to “extremely” is administered to patients to assess their mood states. The BDI and POMS will be assessed at the baseline period, the third menstrual cycle during treatment and the third menstrual cycle in the follow-up period.

#### Patient satisfaction

The Patient Global Impression of Change (PGIC) [19] will be used to assessed patients’ satisfaction for their changes (improvement or decline) in clinical status. The PGIC is a single-item questionnaire used to assess improvement with therapy. A 7-point scale ranging from “very much better” to “very worse” is used to assess their satisfaction states. The PGIC will be assessed at the baseline period, the third menstrual cycle during treatment and the third menstrual cycle in the follow-up period.

#### Pain diary

Pain diaries will be used to record the specific time and frequency of pain attacks, the duration of pain, the changes of intensity, the start time of pain relief and any medication used for patients. Pain diaries will be assessed at the baseline period, the first, second and third menstrual cycles and at follow-up.

### Follow-up

The goal of follow-up is to evaluate the long-term efficacy of acupuncture on pain relief. The follow-up assessments will be performed after treatment and will last for 3 months. During the follow-up period, patients will continue to maintain pain diaries and complete the questionnaires. They can submit the relevant information to researchers via email or short message service. Researchers will also pay close attention to changes in patient illness.

### Randomization and blinding

One hundred and six eligible patients will be randomly assigned to either the treatment group or the control group in a 1:1 ratio after all baseline assessments. The random allocation sequence was generated by a computer. A researcher who does not participate in treatment, data collection and evaluation will provide the random number and group assignment to each center. The randomization is blinded and allocation is hidden. Owing to the unique nature of the acupuncture operation, it is impossible for the acupuncturists to be blinded. However, patients, the data collector and the statistician will be blinded.

### Sample size calculations and statistical analysis

This trial will use a clinical-superiority design that aims to verify that the curative effect of acupuncture is better than that of comfort needles. The primary outcome measure is proposed to be the VAS. The minimal important difference (MID) for improvement on the VAS was found to be − 3.9 cm and/or − 49% [20]. According to our pre-experimental treatment of women with pain related to endometriosis, assuming the standard deviation (SD) to be 1.7 and the difference between two treatment effect to be 3, the statistical power is 80% and the significance level is 0.05. According to the formula [21]:

$$ N=2\times {\left[\left({Z}_{1-\alpha }+{Z}_{1-\beta}\right)/\left(\delta -{\delta}_0\right)\right]}^2\times {S}^2, $$

where *N* represents size per group, *Z*_1-α_ is Type I error, *Z*_1-β_ is Type II error, *δ* is expected SD of the population being sampled, *δ*_0_ is tolerable error in distance from mean value and *S*^2^ is SD. So it requires each group to contain no less than 44 patients. With an estimated 20% dropout rate, we plan to recruit 53 patients for each group, for a total of 106 patients.

Data will be summarized with counts for categorical variables present as mean and SD for normally distributed variables, or median (interquartile) for the variables not normally distributed. Missing data will be imputed according to the last-observation-carried-forward (LOCF) [22] principle. The data will be subjected to intent-to-treat analysis. A statistician blinded to the group settings will perform the statistical analysis. The between-group difference will be compared by analysis of covariance. The variables not normally distributed will be assessed by the rank-sum test. If participants have taken analgesics during the trial, they will be excluded from the analysis and the rate difference of outcomes between the two groups will be analyzed. Statistical significance will be set at *P* < 0.05.

### Data entry and quality control of data

All clinical data will be recorded in the case report form. Researchers must fill in the case report form. Any corrections will be explained in the appended notes signed and dated by the physicians. Supervisors will check each case report form to make sure that the trial data are recorded in a timely manner, accurately and completely. Data input will be done separately by two data collectors with computers. The data manager will examine the data in the database to confirm that it is consistent with the data in the case report form and run a database consistency check.

### Security

Possible adverse events due to acupuncture, such as discomfort, sweating, hematoma, fainting, severe pain, and metal allergy symptoms, will be recorded by the researcher.

## Discussion

This trial is a randomized, multi-center, placebo-controlled clinical trial and aims to demonstrate the efficacy of acupuncture as pain relief in patients with pelvic pain related to endometriosis. When acupuncturists twirl, lift and thrust needles to obtain *De Qi* in the treatment group, patients will have a feeling of burning, numbness, swelling and pain. These are normal physiological reactions [23]. It is also very important to choose the proper time for acupuncture, and we choose to initiate the intervention the week before the expected menstrual onset, three times a week. This can regulate *Qi* and blood [24], to achieve the goal of pain reduction. During the menstrual period, patients will receive acupuncture every day, when they are having pain, in order to alleviate the pain.

In addition, our study also includes an assessment of quality of life, and this is a strength of the present study. Increasing numbers of studies have shown that most patients with pelvic pain related to endometriosis have depression and anxiety, which influence their quality of life [25, 26].

### Trial status

This trial is currently in the recruitment phase.

## Additional file

Additional file 1: Standard Protocol Items: Recommendations for Interventional Trials (SPIRIT) 2013 Checklist: recommended items to address in a clinical trial protocol and related documents. (DOC 111 kb)

## Abbreviations

BDI: Beck Depression Inventory
EHP-30: Endometriosis Health Profile-30
MPI: Multidimensional Pain Inventory
PGIC: Patient Global Impression of Change
POMS: Profile of Mood States
TCM: Traditional Chinese Medicine
VAS: Visual analog scale

## References

[1] Giudice LC (2010). Clinical practice. Endometriosis. N Engl J Med.

[2] Moradi M, Parker M, Sneddon A, Lopez V, Ellwood D (2014). Impact of endometriosis on women’s lives: a qualitative study. BMC Womens Health.

[3] Morotti M, Vincent K, Becker CM (2017). Mechanisms of pain in endometriosis. Eur J Obstet Gynecol Reprod Biol.

[4] Guo SW (2009). Recurrence of endometriosis and its control. Hum Reprod Update.

[5] Sinaii N, Cleary SD, Younes N, Ballweg ML, Stratton P (2007). Treatment utilization for endometriosis symptoms: a cross-sectional survey study of lifetime experience. Fertil Steril.

[6] Langevin HM, Yandow JA (2002). Relationship of acupuncture points and meridians to connective tissue planes. Anat Rec.

[7] Wang SM, Kain ZN, White P (2008). Acupuncture analgesia: I. The scientific basis. Anesth Analg.

[8] Kim SY, Lee H, Chae Y, Park HJ, Lee H (2012). A systematic review of cost-effectiveness analyses alongside randomised controlled trials of acupuncture. Acupunct Med.

[9] Chung YC, Chen HH, Yeh ML (2012). Acupoint stimulation intervention for people with primary dysmenorrhea: Systematic review and meta-analysis of randomized trials. Complement Ther Med.

[10] Smith CA, Zhu X, He L, Song J (2011). Acupuncture for primary dysmenorrhoea. Cochrane Database Syst Rev.

[11] Zhu X, Hamilton KD, Mcnicol ED (2011). Acupuncture for pain in endometriosis. Cochrane Database Syst Rev.

[12] Brown J, Farquhar C (2014). Endometriosis: an overview of Cochrane Reviews. Cochrane Database Syst Rev.

[13] Gui-Wen WU, Zhang P, Jing LI, Wang P, Lin C, Ni-Juan HU (2016). Effect of acupuncture at *Sanyinjiao* (SP6) on the infrared temperature of *Guanyuan* (CV4) and *Sanyinjiao* in dysmenorrhea patients. Shanghai J Acupunct Moxibustion.

[14] Kremer E, Atkinson JH (1981). Pain measurement: construct validity of the affective dimension of the McGill Pain Questionnaire with chronic benign pain patients. Pain.

[15] Jones G, Kennedy S, Barnard A, Wong J, Jenkinson C (2001). Development of an endometriosis quality-of-life instrument: The Endometriosis Health Profile-30. Obstet Gynecol.

[16] Kerns RD, Turk DC, Rudy TE (1985). The West Haven-Yale Multidimensional Pain Inventory (WHYMPI). Pain.

[17] Beck A, Steer R, Brown G. Beck Depression Inventory-II. 1996,490–498.San Antonio: The Psychological Corporation. https://doi.org/10.1093/ndt/gfr086.

[18] Mcnair D, Lorr M, Droppleman L. Manual for the Profile of Mood States. 1971. https://scholar.google.de/scholar?q=mcnair+profile&btnG=&hl=de&as_sdt=0%2C5#1.

[19] Ferguson L, Scheman J (2009). Patient Global Impression of Change scores within the context of a chronic pain rehabilitation program. J Pain.

[20] Wickström K, Edelstam G. Minimal clinically important difference for pain on the VAS scale and the relation to quality of life in women with endometriosis. Sex Reprod Healthc. 2017,13:35–40. 10.1016/j.srhc.2017.05.004 28844356

[21] Zhong B (2009). How to calculate sample size in randomized controlled trial?. J Thorac Dis.

[22] Hamer RM, Simpson PM (2009). Last observation carried forward versus mixed models in the analysis of psychiatric clinical trials. Am J Psychiatr.

[23] Macpherson H, Asghar A (2006). Acupuncture needle sensations associated with *De Qi*: a classification based on experts' ratings. J Altern Complement Med.

[24] Rong P, Zhu B, Li Y, Gao X, Ben H, Li Y (2011). Mechanism of acupuncture regulating visceral sensation and mobility. Front Med.

[25] Morotti M, Vincent K, Brawn J, Zondervan KT, Becker CM (2014). Peripheral changes in endometriosis-associated pain. Hum Reprod Update.

[26] Lumley MA, Cohen JL, Borszcz GS, Cano A, Radcliffe AM, Porter LS (2011). Pain and emotion: a biopsychosocial review of recent research. J Clin Psychol.

